# Olfactory Neuroblastoma Treated by Endoscopic Surgery Followed by Combined External Beam Radiation and Gamma Knife for Optic Nerve and Chiasm Sparing: A Case Report

**DOI:** 10.1155/2011/765645

**Published:** 2011-11-09

**Authors:** Hansi Z. Jiang, Ameer L. Elaimy, Guy C. Jones, Alexander R. Mackay, Robert K. Fairbanks, Wayne T. Lamoreaux, John J. Demakas, Barton S. Cooke, Christopher M. Lee

**Affiliations:** ^1^Gamma Knife of Spokane, 910 W 5th Avenue, Suite 102, Spokane, WA 99204, USA; ^2^University of Washington School of Medicine, 1959 N.E. Pacific Street, Seattle, WA 98195, USA; ^3^Department of Natural Sciences, Carroll College, 1601 N Benton Avenue, Helena, MT 59625, USA; ^4^Cancer Care Northwest, 910 W 5th Avenue, Suite 102, Spokane, WA 99204, USA; ^5^Providence Sacred Heart Medical Center, 101 W 8th Avenue, Spokane, WA 99204, USA; ^6^MacKay & Meyer MDs, Spokane, WA 99202, USA; ^7^Spokane Brain & Spine, Spokane, WA 99204, USA

## Abstract

We describe the multimodality treatment regimen of a 53-year-old man diagnosed with olfactory neuroblastoma (Kadish stage C) in the right nasal cavity extending into the ethmoid sinus and across the cribriform plate. Endoscopic surgery for tumor resection was followed by a combination of external beam radiotherapy and stereotactic radiosurgery boost with concurrent chemotherapy. The novel combination of dual radiation therapies allowed for the preservation of the nearby optic structures while providing an adequate dosage to a sufficient volume of the afflicted tissue.

## 1. Introduction

Olfactory neuroblastoma (ONB) is a rare, malignant tumor of the olfactory epithelium. ONB has been reported in approximately 1000 cases since its first description in 1924. Because of its rarity, definitive treatment guidelines have been limited in scope and depth, but there are still effective treatments for ONB. En-bloc resection combined with radiotherapy has been described as the standard of care [1–3]. Recent studies have shown positive outcomes for tumors treated with endoscopic surgery and adjuvant stereotactic radiosurgery as a minimally invasive treatment modality [4–6]. We present a case of a high-grade ONB near the optic structures treated with a modified combination therapy, endoscopic surgery followed by conventional radiotherapy with radiosurgery boost and concurrent chemotherapy.

## 2. Case Presentation

In February 2009, a 53-year-old man presented to an ENT clinic with increased nasal congestion, increased ear plugging, postnasal drainage, bifrontal headaches, and nasal crusting. A paranasal sinus CT scan showed a deviation of the nasal septum to the left, opacity in the right frontal and anterior ethmoids, and a mass in the right superior meatus. Nasal endoscopy confirmed the presence of an irregular reddish mass in the right nasal cavity. The biopsy showed nests of uniform cells with high nuclear-to-cytoplasmic ratio, round to ovoid nuclei, and fine chromatin. Synaptophysin immunostaining revealed cytoplastic positivity in 100% of the tumor cells, while chromogranin was negative. The patient was diagnosed with ONB and referred to endoscopic surgery.

An MRI scan revealed a 15 × 23 × 46 mm heterogeneously enhancing mass within the right nasal cavity extending into the right ethmoid sinus and crossing the cribriform plate into the right anterior cranial fossa. The mass remained extra-axial. A maxillofacial CT showed a soft tissue mass within the medial right nasal cavity extending superiorly into the ethmoid air cells and crossing the cribriform plate. The tumor was staged at Kadish C with no lymph node metastasis.

In March 2009, the patient underwent a bilateral functional endoscopic sinus surgery: bilateral maxillary antrostomies, bilateral ethmoidectomies, right sphenoidotomy, right frontal sinusotomy, and excision of the right nasal mass. Negative margins were not able to be attained. The patient was then referred to postoperative radiotherapy.

In April 2009, MRI and PET/CT imaging showed no evidence of gross residual disease within the nasal cavity, but there was still concern of microscopic tumor cells. Adjuvant radiation therapy with concurrent cisplatin weekly at 30 mg/m^2^ was prescribed. A standard postoperative radiation dose for ONB is 54–66 Gy, but the tolerance of the optic nerve, the optic chiasm, and the brainstem is limited to 54 Gy. Because of the tumor bed location, damage to the optic tract and chiasm would have been likely to occur if standard radiotherapy doses were prescribed, and radiosurgery alone could not be safely targeted to the full area of involvement. Thus, daily intensity-modulated radiation therapy (IMRT) to the larger area of microscopic disease concern was utilized along with a Gamma Knife (GK) radiosurgery boost to the central tumor bed. IMRT was prescribed at 50 Gy over 25 treatments. The GK prescription of 8.0 Gy to the 50% isodose line resulted in 100% coverage of the target at 8 Gy with excellent dose falloff to the nearby optic structures. A total 58 Gy (biologically equivalent to 66 Gy) was delivered to the tumor bed and 50 Gy to the surrounding areas of concern for subclinical disease. 

At last known followup, twenty-one months after treatment, the patient reported no neurological deficits and no reduction in vision. A CT scan revealed a small, postoperative defect in the medial aspect of the cribriform plate. This was unchanged from prior scans that showed decreased soft tissue within the ethmoid cavity adjacent to the defect within the floor of the anterior cranial fossa. No evidence of pathologic enhancement or progressive soft tissue masses was observed. The patient was clinically well, aside from some mild short-term memory loss and occasional self-limiting headaches.

## 3. Discussion

Determining the optimal treatment regimen for ONB is difficult; the location of a tumor can complicate treatments by limiting the opportunity for resection and radiotherapy because of infiltration into nearby critical structures. Previously regarded treatments include combinations of open surgery, chemotherapy, and radiotherapy. Since the advent of endoscopic surgery for ONB, equal or better outcomes for patients undergoing combined endoscopic surgery and GK radiosurgery have been observed [4–8]. When comparing endoscopic surgery to open surgery, a greater survival rate has been found for endoscopic techniques although the study did not distinguish among adjuvant treatments [6]. However, the scope of this treatment can sometimes be limited. For example, tumor invasion into the anterior cranial fossa indicates an anterior craniotomy since endoscopic procedures are inadequate in this area [9]. Thus, combined endoscopic surgery and stereotactic surgery treatments are usually confined to patients with early stage tumors [6]. But advances in endoscopic tools and techniques have extended the reach of endoscopic procedures, as demonstrated in this report.

We present a unique case of curative therapy with vision preservation by utilizing Gamma Knife therapy as a radiation boost. The unfortunate location of the patient's tumor fostered a need for creative treatments in order to preserve vision while also maintaining high local tumor control. The endoscopic procedure followed by combination radiation therapy (consisting of IMRT and GK boost) is a unique regimen that treats the tumor and also preserves nearby optic structures. IMRT targets a wider area around the tumor bed while staying under the radiation tolerance of the nearby optic structures; the GK boost provides the focal dose escalation to the tumor bed with excellent dose falloff to the sensitive structures. Either IMRT or GK alone could not have avoided these sensitive structures as effectively or provided the necessary volume coverage.
